# Effect of thong style flip-flops on children’s barefoot walking and jogging kinematics

**DOI:** 10.1186/1757-1146-6-8

**Published:** 2013-03-05

**Authors:** Angus Chard, Andrew Greene, Adrienne Hunt, Benedicte Vanwanseele, Richard Smith

**Affiliations:** 1 Discipline of Exercise and Sport Science, Faculty of Health Science, The University of Sydney, Sydney, NSW, 2006, Australia; 2 Postgraduate Medical Institute, Faculty of Health, Social Care and Education, Anglia Ruskin University, Chelmsford, UK; 3 Human Biomechanics Research Group, Department of Kinesiology, KU Leuven, Belgium/ Health, Innovation & Technology, Fontys University of Applied Sciences, Eindhoven, Netherlands

## Abstract

**Background:**

Thong style flip-flops are a popular form of footwear for children. Health professionals relate the wearing of thongs to foot pathology and deformity despite the lack of quantitative evidence to support or refute the benefits or disadvantages of children wearing thongs. The purpose of this study was to compare the effect of thong footwear on children’s barefoot three dimensional foot kinematics during walking and jogging.

**Methods:**

Thirteen healthy children (age 10.3 ± 1.6 SD years) were recruited from the metropolitan area of Sydney Australia following a national press release. Kinematic data were recorded at 200 Hz using a 14 camera motion analysis system (Cortex, Motion Analysis Corporation, Santa Rosa, USA) and simultaneous ground reaction force were measured using a force platform (Model 9281B, Kistler, Winterthur, Switzerland). A three-segment foot model was used to describe three dimensional ankle, midfoot and one dimensional hallux kinematics during the stance sub-phases of contact, midstance and propulsion.

**Results:**

Thongs resulted in increased ankle dorsiflexion during contact (by 10.9°, *p;* = 0.005 walk and by 8.1°, *p;* = 0.005 jog); increased midfoot plantarflexion during midstance (by 5.0°, *p;* = 0.037 jog) and propulsion (by 6.7°, *p;* = 0.044 walk and by 5.4°, *p;*= 0.020 jog); increased midfoot inversion during contact (by 3.8°, *p;*= 0.042 jog) and reduced hallux dorsiflexion during walking 10% prior to heel strike (by 6.5°, *p;* = 0.005) at heel strike (by 4.9°, *p;* = 0.031) and 10% post toe-off (by 10.7°, *p;* = 0.001).

**Conclusions:**

Ankle dorsiflexion during the contact phase of walking and jogging, combined with reduced hallux dorsiflexion during walking, suggests a mechanism to retain the thong during weight acceptance. Greater midfoot plantarflexion throughout midstance while walking and throughout midstance and propulsion while jogging may indicate a gripping action to sustain the thong during stance. While these compensations exist, the overall findings suggest that foot motion whilst wearing thongs may be more replicable of barefoot motion than originally thought.

## Background

Thongs (also known as flip-flops) are a common footwear choice for Australian children [1]. They are typically constructed from a rubber template which is loosely secured to the foot by a single V-shaped rubber strap extending from between the first web space to the base of the first and fifth metatarsals. Footwear is regarded as necessary apparel for foot comfort and protection. Due to their flexible and unrestrictive nature, thongs may be preferable to other children’s footwear types, all of which have been shown to alter natural foot function [2], since the ideal footwear for a child’s developing feet is believed to be that which allows natural motion of the foot [3,4]. In support of this view are reports that, compared to habitually shod children, habitually unshod children have stronger and healthier feet with less incidence of toe deformity [3].

Despite the possible advantage of thongs compared to other footwear options for children, there is no evidence that they are beneficial. Indeed, there are concerns that thongs may be harmful. In a recent survey of 272 parents of children, thongs were implicated by the parents as contributing to 15% of forefoot and 22% of rearfoot complaints [1]. Prolonged use of thongs has been linked to heel pain [5] and shin-splints [6]. However, there exists no empirical evidence to explain the mechanisms for specific pathologies, and no analysis of the effect of thong wearing on foot function in children. From studies of adults, thongs have been found to result in increased ankle plantarflexion at heel contact, compared to sneakers [7] and decreased plantar pressure at the rearfoot, forefoot and hallux, compared with barefoot [8]. Whilst the implications of these findings are unclear, the cushioning effect of a thong indicated by the decreased pressure challenges the commonly held belief of the need to claw the toes in order to maintain interaction between the barefoot and the thong. Other pathological mechanisms that are concerning because of their associations with symptoms in adults and may potentially occur in children who wear thongs include; that of plantar fasciitis with flattening of the longitudinal arch [9]; and foot pronation and reduced hallux dorsiflexion [10]; and medial tibial stress syndrome also known as shin-splints with excessive foot pronation [11] and rearfoot eversion [12]. However, there have been no studies of the effects on foot function in children to support or refute any concerns or harm in wearing thongs.

To our knowledge, no quantitative evidence to support or refute the benefits or disadvantages of children wearing thongs has been reported in the literature. The aim of this paper is to compare the kinematic effects of wearing thongs on children’s feet with a barefoot control condition during walking and jogging using three dimensional motion analyses. It is hypothesised that, compared to barefoot, wearing thongs will see reduced hallux motion, greater midfoot dorsiflexion and ankle eversion.

## Methods

### Participants

Study participants were thirteen children (8 girls and 5 boys) between 8 and 13 years of age (mean age 10.3 ± 1.6 SD years) from the metropolitan area of Sydney Australia who volunteered in response to publicly displayed posters, press release and fourteen radio interviews. Power analysis using data from a previous footwear study [13] indicated that twelve participants would be necessary to achieve a significant difference with alpha set at 0.05 and power set at 0.8 with the effect size 0.62. This number is similar to Leardini et al’s [14] protocol for measuring multi segment foot motion, which found meaningful differences with ten participants.

Inclusion criteria stipulated healthy children free of known foot deformity, and not requiring medical consultation for foot or leg pathology in the preceding six months, Beighton Score less than 5/9 to exclude hypermobile children [15] and a foot posture index (FPI) within 2 SD of normal to exclude excessively pronated and supinated foot types [16]. The University of Sydney Human Ethics Committee granted ethics approval for this study and a parent/carer of each participant gave written consent together with the child’s informed verbal assent prior to participation.

### Model, segment and joint angle definitions

**Figure 1:**
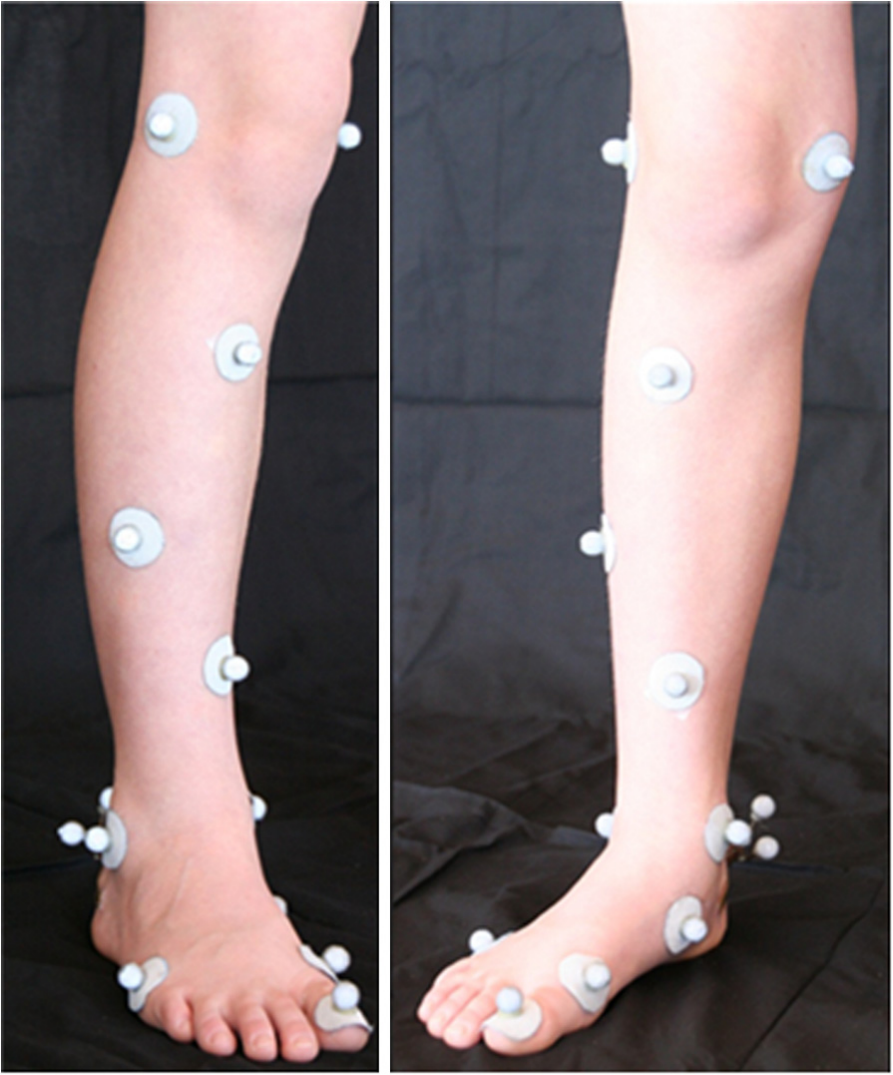
Images of the foot and leg with markers used for the definition of segments and their embedded axes.

Shank and rearfoot segments were defined using 3, 12 mm diameter, non-collinear reflective markers per segment (Figure 1). Motion of the shank was determined using markers placed on areas of minimal soft tissue movement at the proximal, distal and lateral shank. Motion of the rearfoot was determined using a detachable wand triad marker previously shown to be valid and reliable [17]. It consisted of an array of three markers mounted onto a rigid shaft that attached to the calcaneus via a flexible metal stirrup. The stirrup provided a large contact area around the calcaneus and was secured using double sided adhesive tape and strapping tape. Motion of the forefoot was determined with markers located at the navicular, first and fifth metatarsal heads. The first metatarsal segment was defined by the line from the navicular to first metatarsal head and the hallux segment by the line from the first metatarsal head to the marker located dorsal to first distal phalanx.

The two joints of the rearfoot (talocrural and subtalar) were considered as a single universal joint with its centre located at the midpoint between the markers on the medial and lateral malleolus. The forefoot segment X and Y axes had their origin in line with the navicular marker and the Z axis in line with the rearfoot joint centre. The axis system origin for the shank segment was midway between the medial and lateral femoral condyles. All segment X axes were initially aligned with the laboratory -Z axis (down), segment Y axis pointing anteriorly (X axis of laboratory) and the segment Z axis pointing to the right of the participant (-Y axis of the laboratory). For the shank segment the X axis was subsequently aligned with the rearfoot joint centre.

The three degrees of freedom ankle joint angle was described using the joint coordinate system according to International Society of Biomechanics (ISB) recommendations [18]. The midfoot angle describing the angular relationship between the forefoot and the rearfoot used a similar joint coordinate system as that for the ankle. That is, the midfoot plantarflexion/dorsiflexion axis was the Z-axis of the rearfoot, the midfoot abduction/adduction axis was the X-axis of the forefoot and the inversion/eversion axis of the midfoot was the cross product between the Z-axis of the rearfoot and the X-axis of the forefoot.

### Experimental approach

**Figure 2:**
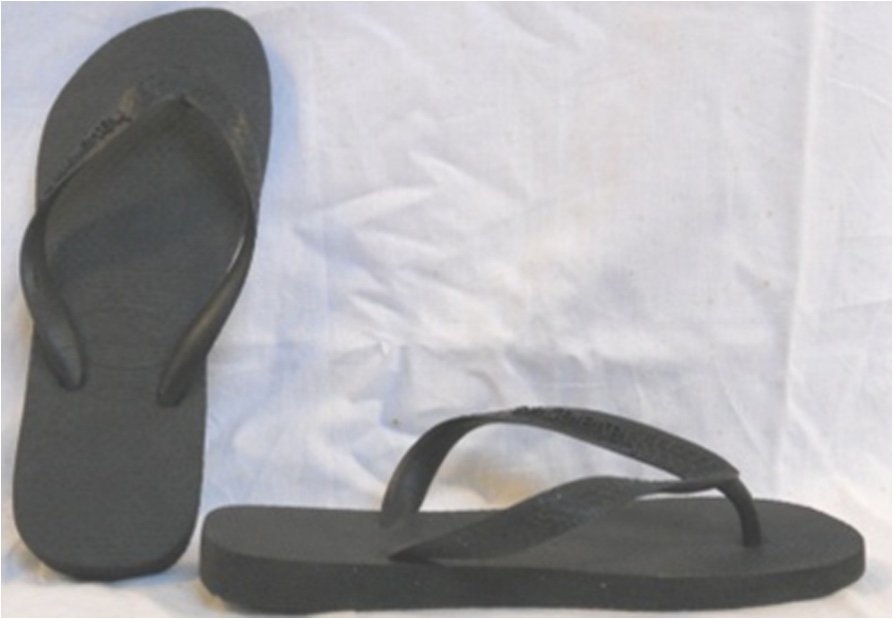
Example simple non-contoured thongs.

Study participant characteristics were recorded (Table 1) and reflective markers applied prior to a standardised foot reference position being recorded. Participants practised walking and jogging along the seven metre walkway at a self-selected pace while visually attending to a distant line bisecting the lab to maintain direction and avoid targeting of the force plate. Participants then conducted five walking trials and five jogging trials in a straight line while barefoot, or while wearing simple, non-contoured thongs (Figure 2). The test order of barefoot and a thong condition was randomised between participants.

**Table 1:** Study participant characteristics (n = 13)

**Variable**	**Mean or count**	**Range**
Gender, male:female	5:8	NA
Age, years (SD)	10.3(1.6)	8 - 13
Height, m (SD)	1.4 (0.1)	1.2 - 1.6
Body mass, kg (SD)	34.0 (8.2)	21.6 - 47.8
Beighton Score (SD)	2.7 (1.6)	0-4
Foot Posture Index, score (SD)	6 (0.1)	2 - 9
Dominant leg, right (%)	12 (92)	NA
Thong size	35/36	31/32 – 39/40

### Equipment

Video data were recorded at 200 Hz using a 14 camera motion analysis system (Cortex Version 1.1, Motion Analysis Corporation, Santa Rosa, USA). The initial right foot ground reaction force was measured using a force platform (Model 9281B, Kistler, Winterthur, Switzerland). Calibration of all fourteen cameras was completed prior to each session of data collection. Residual error for the motion analysis system, representing the accuracy with which the system could reconstruct marker location within the captured volume, was <0.5mm across all testing sessions.

### Data processing

All trials were truncated at 20% prior to heel-strike of the right foot and at 20% after the right foot toe-off and time normalised to the right foot’s stance phase. In accordance with previous literature, the kinematic data were smoothed at 5 Hz [19] for walking and 20 Hz [20] for jogging. Relative angles were calculated using KinTrak software (University of Calgary, Canada). The timing of heel contact and toe-off events was established from the vertical ground reaction force. For each participant and condition the mean of five trials was calculated. The ensemble mean and 95% confidence intervals across participants were computed. The confidence intervals were used to determine whether differences were significant between conditions for the continuous data.

Four events were used to define the three stance sub-phases: foot contact (heel contact to foot flat), mid-stance (foot flat to heel rise) and propulsion (heel rise to toe off). Foot flat and heel rise events were defined within stance phase using the minimum of the posteriorly directed and the zero-crossing of the anterior-posterior ground reaction force respectively.

### Statistical analysis

For the primary discrete variable of the footwear condition thong to barefoot, a two by five nested repeated measures analysis of variance was used (SPSS Version 19, IBM SPS Inc, USA). Bonferoni adjustments to condition and gait were applied to test significant differences between footwear conditions and gaits walking and jogging over five trials. The threshold of *p* < 0.05 was set to determine the significance of range of motion value and mean difference.

## Results

### Participants

Mean age, height, mass, Beighton Score, FPI, dominant leg and thong size for the participants are presented in Table 1. On average, during walking in barefoot and thong conditions, foot-flat occurred at 13 and 14 percent of stance and heel-rise at 54 and 53 percent of stance respectively_._ During jogging, barefoot and in thong conditions, foot-flat occurred at 20 and 22 percent of stance and heel-rise at 44 and 44 percent of stance respectively_._ Participant barefoot and thong ankle, midfoot and hallux range of motion (ROM) and walking and jogging velocity are shown in Table 2.

**Table 2:** **Mean, *p *value and 95% confidence interval for the difference between the means for the joint range of motion and velocity over the stance phase for barefoot and thong while walking and jogging**

	**Walk**						**Jog**					
**Variable**	**Barefoot**		**Thong**				**Barefoot**		**Thong**			
	***Angle (°)***	***SD***	***Angle (°)***	***SD***	***p;<0.05***	***95% CI***	***Angle (°)***	***SD***	***Angle (°)***	***SD***	***p;<0.05***	***95% CI***
Ankle sagittal	22.4	5.4	20.7	7.9	0.386	−2.39, 5.76	28.2	5.4	25.8	5.8	0.085	−0.383, 5.18
Ankle frontal	11.6	2.5	11.1	2.1	0.580	−1.59, 0.940	12.8	3.2	12.5	2.5	0.626	−1.03, 1.64
Ankle transverse	12.1	3.6	12.6	4.3	0.688	−2.78, 1.90	10.1	3.6	9.3	3.2	0.162	−0.376, 2.01
Midfoot sagittal	21.8	6.1	21.2	6.1	0.701	−2.69, 3.87	25.0	5.0	22.9	3.2	0.116	−0.589, 4.69
Midfoot frontal	7.0	1.4	7.4	4.0	0.656	−2.51, 1.64	6.7	3.2	6.0	2.2	0.452	−1.34, 2.82
Midfoot transverse	7.5	3.2	8.4	3.2	0.099	−1.90, 0.186	5.9	1.8	5.2	1.8	0.327	−0.794, 2.20
Hallux sagittal	30.4	7.6	25.3	6.5	0.80	−1.42, 5.29	25.0	7.6	22.3	6.1	0.170	−1.30, 6.56
Mean velocity (ms^-2^)	1.4	0.2	1.4	0.2	0.079	−1.24, 1.56	2.5	0.2	2.5	0.2	0.520	−2.34, 2.66

* indicates a significant difference compared to barefoot when p < 0.05.

### Kinematics

#### Walking

**Figure 3:**
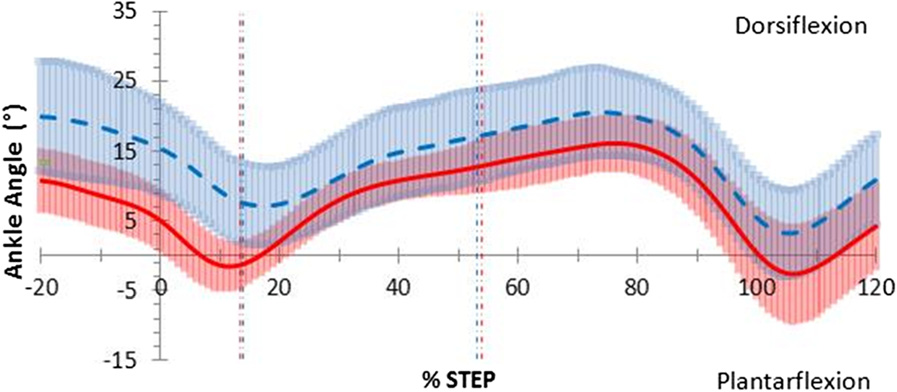
**Sagittal plane ankle motion in walking gait.** Mean joint angles for barefoot (red) including 95% confidence intervals (red shading) and thong (blue dashed) including 95% confidence intervals (blue shading), including 20% before and 20% after stance (y-axis), while walking. Events foot-flat and heel-rise represented by the vertical red (barefoot) and blue (thong) dashed lines.

At heel strike the ankle was 10.4°, (*p*; = 0.010, 95% CI [2.02, 18.73]) more dorsiflexed in the thong condition when compared to barefoot (Figure 3) and remained more dorsiflexed by 10.9°, (*p;* = 0.005, 95% CI [−4.04, 17.75]) throughout the contact phase (Figure 3). Over the entire stance phase, the ankle averaged greater dorsiflexion in the thong condition, although this difference was not significantly different at 5.3°, (*p;* = 0.122, 95% CI [−1.66, 12.32]). Ankle frontal and transverse plane motion in the thong condition closely followed that of barefoot throughout the stance phase.

**Figure 4:**
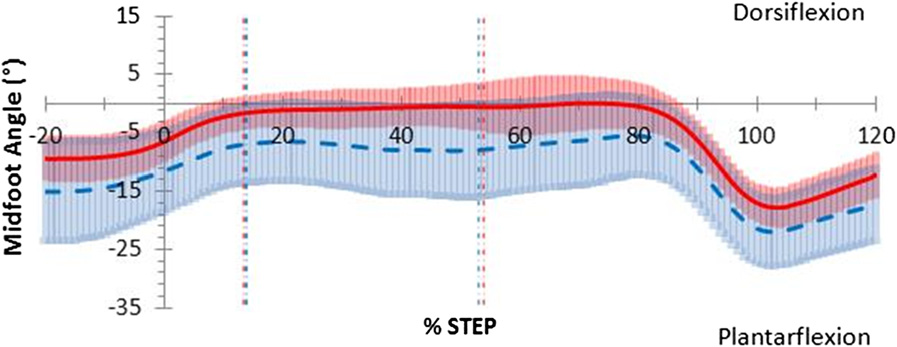
**Sagittal plane midfoot motion in walking gait.** Mean joint angles for barefoot (red) including 95% confidence intervals (red shading) and thong (blue dashed) including 95% confidence intervals (blue shading), including 20% before and 20% after stance (y-axis), while walking. Events foot-flat and heel-rise represented by the vertical red (barefoot) and blue (thong) dashed lines.

The pattern of midfoot sagittal plane motion (Figure 4) was similar between barefoot and thong conditions, although the thong condition demonstrated a trend towards increased plantarflexion throughout stance by 6.5°, (*p;* = 0.055, 95% CI [−0.156, 13.3]) (Table 3). Although not significant the following two observations were noted. During the contact phase, when thongs were worn, the midfoot was more plantarflexed by 5.4° (*p;* = 0.090, 95% CI [−1.00, 11.9]) than when barefoot. During mid-stance, the midfoot was more plantarflexed by 6.6°, (*p;* = 0.052, 95% CI [−0.065, 13.3]) when thongs were worn. The midfoot was more plantarflexed when thongs were worn during the propulsive phase by 6.7°, (*p;* = 0.044, 95% CI [0.205, 13.3]) (Table 3). Midfoot frontal and transverse plane motion in the thong condition showed no difference to barefoot through the stance phase (Table 3).

**Table 3:** **Mean, *p *value and 95% confidence interval for the difference between the angle means over the stance phase for barefoot and thong while walking and jogging**

			***WALK***				***JOG***			
**Phase**	**Joint**	**Plane**	**Barefoot**	**Thong**	***p;<0.05***	**95% CI**	**Barefoot**	**Thong**	***p;<0.05***	**95% CI**
**Contact**	**Ankle**	Sagittal	1.1(8.3)	12.0(12.2)	0.005*	−17.8, -4.04	13.0(7.6)	21.1(10.4)	0.005*	−13.2, -2.98
		Frontal	−7.5(3.6)	−10.0(5.8)	0.694	−3.23, 2.25,	−4.7(4.3)	−3.6(4.7)	0.31	−3.30, 1.14
		Transverse	1.6(4.7)	1.7(3.2)	0.957	−3.15, 3.00	3.6(5.0)	1.4(5.8)	0.194	−1.30, 5.77
	**Midfoot**	Sagittal	−3.8(6.5)	−9.2(12.2)	0.090	−1.00, 11.9	−3.5(6.5)	−6.6(11.9)	0.203	−1.92, 8.13
		Frontal	2.0 (2.9)	0.6 (5.0)	0.181	−0.750, 3.55	1.2(3.6)	−2.6(6.5	0.042*	0.15, 7.40
		Transverse	−3.6(3.6)	1.0(4.0)	0.441	−5.21, 2.42	−0.8(4.3)	2.2(4.3)	0.075	−6.30, 0.35
	**Hallux**	Sagittal	6.2(4.3)	3.7(5.4)	0.203	−1.57, 6.65	4.3(4.7)	3.3(6.5)	0.694	−4.41, 6.41
**Midstance**	**Ankle**	Sagittal	7.6(5.8)	12.6(10.4)	0.124	−11.44, 1.56	20.3(5.4)	24.4(9.0)	0.099	−9.02, 0.88
		Frontal	−3.8(3.6)	−4.2(4.7)	0.672	−1.47, 2.20	0.3(4.7)	1.0(4.7)	0.492	−2.94, 1.50
		Transverse	4.1(4.3)	2.5(4.0)	0.315	−1.74, 4.96	6.4(5.8)	3.4(5.8)	0.069	−0.28, 6.40
	**Midfoot**	Sagittal	−0.8(6.1)	−7.4(12.6)	0.052	−0.065, 13.3	2.9(7.2)	−2.2(12.6)	0.037*	0.370, 9.73
		Frontal	3.6 (1.8)	2.2 (5.0)	0.231	−1.02, 3.83	2.0(4.3)	−0.5(6.1)	0.140	−0.908, 5.71
		Transverse	0.5(3.2)	1.9(4.7)	0.421	−5.21, 2.33	0.1(4.7)	2.8(4.0)	0.085	−5.82, 0.435
	**Hallux**	Sagittal	1.1(2.9)	1.2(4.7)	0.926	−2.99, 2.74	−0.3(3.2)	0.4(4.7)	0.664	−4.26, 2.81
**Propulsive**	**Ankle**	Sagittal	12.4(8.3)	16.4(11.5)	0.282	−3.74, 11.8	18.0(6.5)	21.0(10.4)	0.242	−8.12, 2.27
		Frontal	−6.8(5.0)	−6.8(5.0)	0.552	−1.44, 2.56	−3.6(4.3)	−3.0(4.7)	0.536	−2.68, 1.47
		Transverse	0.1(5.4)	−1.3(4.3)	0.383	−1.97, 4.78	3.6(5.8)	0.8(6.1)	0.098	−0.591, 6.09
	**Midfoot**	Sagittal	−3.4(7.2)	−10.1(12.6)	0.044*	0.205, 13.3	−2.1(7.9)	−7.5(11.9)	0.020*	−9.84, -1.01
		Frontal	2.5(1.8)	1.9(4.7)	0.645	−2.07, 3.18	1.8(4.0)	−0.3(5.4)	0.153	−0.908, 5.13
		Transverse	−0.7(3.2)	0.5(4.7)	0.524	−5.00, 2.69	−0.7(4.3)	1.7(3.6)	0.107	−5.37, 0.594
	**Hallux**	Sagittal	7.0(4.3)	6.0(6.8)	0.675	−3.89, 5.81	7.6(5.4)	5.4(4.3)	0.249	−1.77, 6.30
**Stance**	**Ankle**	Sagittal	9.1(6.8)	14.4(8.3)	0.122	−12.32, 1.66	17.6(6.1)	22.0(9.7)	0.070	−9.23, 0.416
		Frontal	−5.5(4.0)	−5.8(5.0)	0.775	−1.68, 2.19	−2.8(4.3)	−2.0(4.3)	0.426	−2.86, 1.29
		Transverse	1.9(4.7)	0.7(4.0)	0.444	−2.11, 4.52	4.3(5.8)	1.7(5.8)	0.120	−0.798, 6.06
	**Midfoot**	Sagittal	−2.5(6.5)	−9.0 (13.0)	0.055	−0.156, 13.3	−1.1(7.2)	−5.6(11.9)	0.051	−0.31, 9.17
		Frontal	2.8(2.2)	1.7(4.7)	0.362	−1.37, 3.48	1.7(4.0)	−0.9(5.8)	0.102	−0.596, 5.78
		Transverse	−0.2(3.2)	1.2(4.3)	0.435	−5.20, 2.39	−0.5(3.6)	2.2(4.0)	0.084	−5.65, 0.409
	**Hallux**	Sagittal	1.1(2.9)	1.2(4.3)	0.926	−2.99, 2.74	4.9(4.3)	3.5(3.2)	0.384	−2.08, 5.08

* indicates a significant difference compared to barefoot when p < 0.05.

Mean joint angles for ankle and midfoot sagittal, frontal and transverse planes and hallux sagittal plan during stance. Positive value indicates dorsiflexion, eversion and abduction; negative values indicate plantarflexion, inversion and adduction.

**Figure 5:**
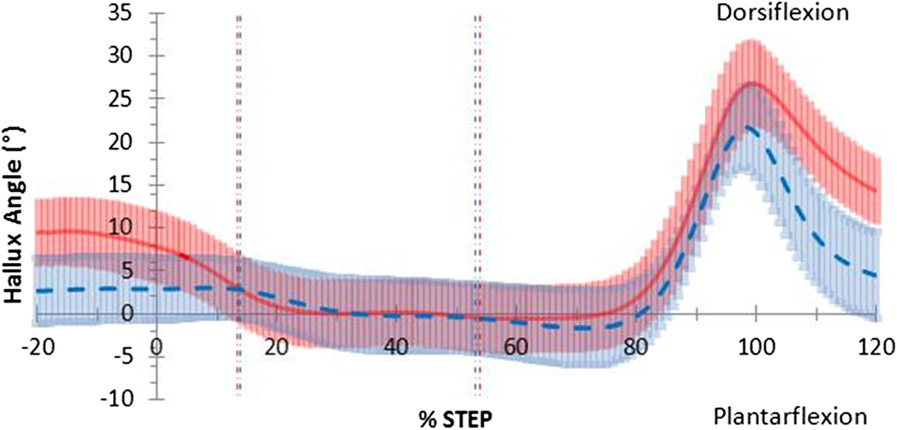
**Sagittal plane hallux motion in walking gait.** Mean joint angles for barefoot (red) including 95% confidence intervals (red shading) and thong (blue dashed) including 95% confidence intervals (blue shading), including 20% before and 20% after stance (y-axis), while walking. Events foot-flat and heel-rise represented by the vertical red (barefoot) and blue (thong) dashed lines.

Hallux sagittal plane position in the thong condition was less dorsiflexed prior to heel strike at −10% of stance by 6.5°, (*p;* = 0.005, 95% CI [3.76, 7.67]) at heel strike by 4.9°, (*p;* = 0.031, 95% CI [3.68, 7.78]) and at 110% of stance by 10.7°, (*p*; = 0.001 95%CI [3.49, 17.93]) (Figure 5).

#### Jogging

**Figure 6:**
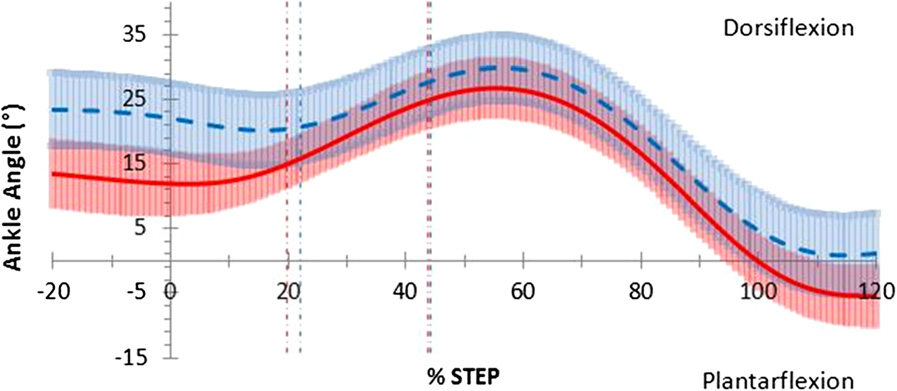
**Sagittal plane ankle motion in jogging gait.** Mean joint angles for barefoot (red) including 95% confidence intervals (red shading) and thong (blue dashed) including 95% confidence intervals (blue shading), including 20% before and 20% after stance (y-axis), while jogging. Events foot-flat and heel-rise represented by the vertical red (barefoot) and blue (thong) dashed lines.

Greater ankle dorsiflexion occurred in the thong condition at heel-strike by 10.2°, (*p*; = 0.003, 95% CI [2.25, 17.74]) and following toe-off at 110% of stance by 5.8°, (*p;* = 0.016, 95%CI [4.69, 6.09]) (Figure 6). Over the entire stance phase, ankle sagittal plane motion when thongs were worn was similar in pattern to barefoot jogging. This occurred despite a trend towards greater dorsiflexion throughout stance when thongs were worn 4.4°, (*p;* = 0.070 95%CI [−0.416, 9.23]) (Table 3). No difference was seen for ankle frontal plane or transverse plane motions when thongs were worn (Table 3).

**Figure 7:**
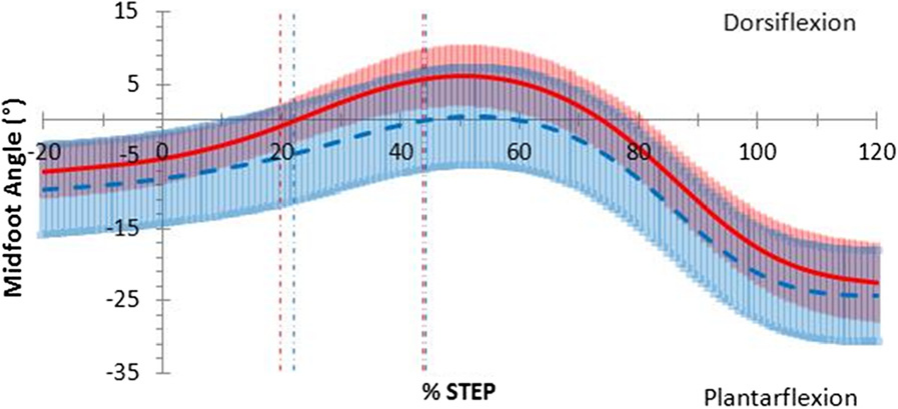
**Sagittal plane midfoot motion in jogging gait.** Mean joint angles for barefoot (red) including 95% confidence intervals (red shading) and thong (blue dashed) including 95% confidence intervals (blue shading), including 20% before and 20% after stance (y-axis), while jogging. Events foot-flat and heel-rise represented by the vertical red (barefoot) and blue (thong) dashed lines.

**Figure 8:**
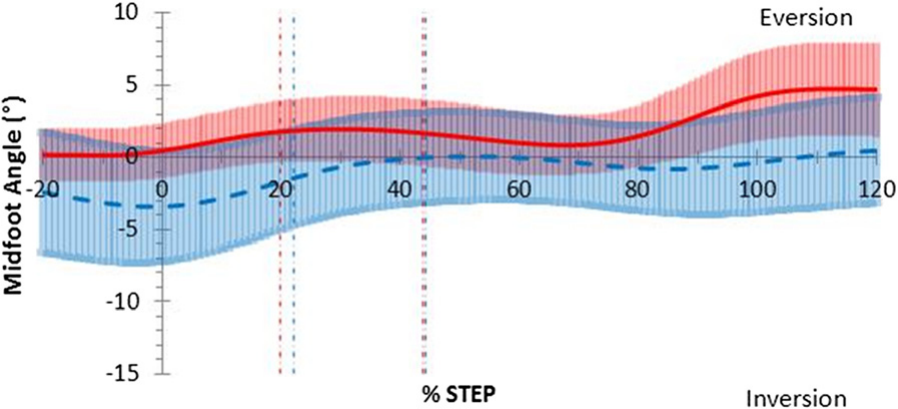
**Frontal plane midfoot motion in jogging gait.** Mean joint angles for barefoot (red) including 95% confidence intervals (red shading) and thong (blue dashed) including 95% confidence intervals (blue shading), including 20% before and 20% after stance (y-axis), while jogging. Events foot-flat and heel-rise represented by the vertical red (barefoot) and blue (thong) dashed lines.

The midfoot was more plantarflexed during the thong condition in the sagittal plane during midstance by 5.0°, (*p;* = 0.037, 95% CI [0.37, 9.73]) and propulsion by 5.4°, (*p;* = 0.020, 95% CI [1.01, 9.84]) (Table 3) (Figure 7). The midfoot mean plantarflexion angle was greater by 4.6° (*p;* = 0.051, 95% CI [−0.031, 9.17]) over the entire stance phase when thongs were worn but not significantly so (Table 3). The midfoot was more inverted during the contact phase by 3.8°, (*p;* = 0.042, 95% CI [0.15, 7.40]) (Figure 8) (Table 3) and at toe-off by 4.6°, (*p;* = 0.008, 95% CI [3.44, 5.65]). Midfoot transverse plane motion in the thong condition showed no difference to barefoot throughout the stance phase (Table 3).

Hallux sagittal plane motion was unaffected by thongs while jogging.

## Discussion

The purpose of this study was to examine the effects of wearing thongs on selected foot kinematics while children were walking and jogging using the barefoot condition as a baseline. Children adapted to wearing thongs with altered ankle kinematics during the contact phase while walking and jogging and midfoot adaptations during midstance while jogging. Hallux adaptations were observed while walking prior to and during weight acceptance and after toe off. Overall ankle, midfoot and hallux range of motion was unaffected while wearing thongs compared to barefoot.

Self-selected barefoot walking velocity (Table 2) in the present study is consistent with previous studies [21,22]. Thongs had a minimal effect on barefoot walking and jogging velocities. Barefoot walking joint angle ROM (Table 2), in the current study are consistent with previously reported literature for those papers that used the relative angle of the shank to the rearfoot to describe sagittal plane ankle ROM in children [22]. Since children’s gait is considered to be mature by age four [23] and foot mechanics mature by age five [24], comparisons can be drawn between the current study and adult studies using the same joint definition models. Consistencies were identified between the current data and adult ankle ROM in the sagittal [19,25,26], frontal [19,25] and transverse planes [19,25] and the midfoot ROM in the sagittal [19,25] and frontal planes [19].

Only small differences were seen when children wore thongs compared to barefoot for the tested foot model. The overall pattern and range of joint angle motion for ankle, midfoot and hallux kinematics were comparable between barefoot and thong conditions during both walking and jogging (Table 2). Barefoot kinematics were altered when thongs were worn throughout various phases of the gait cycle, with these changes mainly occurring in the sagittal plane (Table 3). Participants wearing thongs exhibited more ankle dorsiflexion throughout the contact phase while walking together with midfoot inversion while jogging, more midfoot plantarflexion during midstance while jogging, more midfoot plantarflexion while walking and jogging during the propulsive phase (Table 3) and hallux dorsiflexion was reduced prior to and post stance phase while walking (Figure 5).

Ankle and midfoot adaptations occurred during the contact and midstance phases while wearing thongs compared to barefoot. Significant ankle dorsiflexion during walking (Figure 3) and jogging (Figure 6) combined with midfoot inversion during jogging (Figure 8) prior to and during the contact phase may be a compensatory mechanism necessary to retain thongs on the foot. This increased dorsiflexed and inverted position through loading may have implications for the tibialis anterior muscle, which has been shown through eccentric contraction to be a primary resistor of foot plantarflexion and rearfoot eversion during the first 10% of the stance phase [27]. Previously reported evidence of increased foot plantarflexion seen when wearing thongs compared to shod conditions [7] cannot be directly compared to the current study given their use of a two dimensional single segment foot model in which markers were placed on the outer surface of participants pre-worn shoes, and motion of the rearfoot segment were not measured.

An action to grip thongs may be present during midstance and in particular during propulsion with greater midfoot plantarflexion while walking (Figure 4) and to a greater extent while jogging (Figure 7). The midfoot was more plantarflexed during midstance phase while walking and more plantarflexed during the propulsive phase of walking and jogging (Table 3).

Anecdotally, clinicians have believed it necessary to claw one’s toes to maintain thongs. This popular belief has been found lacking with hallux plantar pressure measures reduced when wearing thongs [8]. The present study confirms this outcome during the stance phase with hallux angular displacement remaining unchanged (Table 2) between conditions while walking (Figure 5) or jogging.

Reduced hallux dorsiflexion immediately prior-to and at heel strike while walking may indicate an action to grip and lever the thong to make contact with the heel in preparation for weight acceptance at heel strike (Figure 5). This adaptation may disrupt tensioning of the plantaraponeurosis with preload, thought to be important for midfoot stability in preparation for load acceptance [28] and increase demand of other midfoot stabilising structures including plantar intrinsic foot muscles [29,30]. Reduced hallux dorsiflexion seen at 110% of stance following toe-off while walking (Figure 5) has implications for hallux clawing during the swing phase of the gait cycle reducing ground clearance, known to be related to trips and falls [31] and thought to be a protective antalgic response of the symptomatic foot [10].

There were a number of limitations to the current study. Firstly, the inclusion criterion was restrictive. This limits the extent to which the findings can be generalized, and cannot be applied to those children with excessively flat or highly arched feet. Further research is required to substantiate the current findings. Our study considered the influence of thongs on children’s kinematics in the immediate time after the thongs were put on and prior wear of thongs was not controlled for. Children were not separated into groups of habitual or infrequent wearers of thongs which may have an effect on condition familiarity and the individual methods to secure the thong. Future studies should include side-stepping tasks and should examine the inverse dynamics during prolonged wearing of thongs to better understand pathological implications of the processes necessary to maintain thongs and their effect.

## Conclusions

Thongs had a minimal effect on walking and jogging at self-selected speed. The adaptations seen in this study may be necessary to maintain contact between the thong and the foot. In particular, increased contact phase ankle dorsiflexion, during walking and jogging with reduced hallux dorsiflexion during walking suggests a need to retain the thong during weight acceptance. Greater midfoot plantarflexion during midstance while jogging and propulsion while walking and jogging suggests a gripping action to retain the thong during stance. Reduced hallux dorsiflexion after toe-off during walking indicates a gripping action may be necessary during early swing. These adaptations may result in muscle overuse syndromes for rearfoot dorsiflexors and midfoot plantarflexors with prolonged thong wear, however further evidence is required to explore these areas. While differences were statistically significant, clinical importance is yet to be determined and so, overall, foot motion whilst wearing thongs may be more replicable of barefoot motion than originally thought.

## Competing interests

The authors declare no competing interests.

## Authors’ contributions

AC carried out participant recruitment, data collection and analysis, statistical analysis, interpretation of results and manuscript draft. AG assisted data collection, statistical interpretation and editing the manuscript. AH and BV assisted in editing the manuscript. RS conceived the study, constructed the methodology, procedure for statistical analysis and interpretation and study overall coordination and helped to edit the manuscript. All authors read and approved the final manuscript.
